# Evaluation of an Environmental Nutrition Intervention at the 2018 Commonwealth Games

**DOI:** 10.3390/nu15214678

**Published:** 2023-11-04

**Authors:** Fiona E. Pelly, Rachael L. Thurecht

**Affiliations:** School of Health, University of the Sunshine Coast, Sippy Downs, QLD 4556, Australia; rthurech@usc.edu.au

**Keywords:** food provision, food environment, major competition, athletes, catering

## Abstract

There has been an increasing expectation that the food provided for athletes at major competition events meets the specific dietary and performance needs of athletes. The aim of this study was to map the range of food service nutrition schemes that were implemented prior to and during a major competition event (2018 Commonwealth Games) and evaluate these schemes through staff training satisfaction, athlete feedback, and quality assurance checks. This study followed a case study design with nutrition schemes as follows: informing (nutrition labelling), enabling (staff training, nutrition service), and engineering (modification to menus and recipes). Overall, participants reported that they easily found items on the menu that met their nutritional/dietary needs. When asked how useful the schemes were in helping them to identify items that meet their needs, the majority of participants found the nutrition cards (*n* = 227, 71%) and serving staff (*n* = 212, 66%) ‘useful/very useful’. ‘Good/very good’ ratings were received by >90% of respondents for speed of service, staff politeness, and knowledge of the menu. Participants (*n* = 316) who rated the nutrition staff as ‘useful/very useful’ gave a higher median rating for the menu. Past events have focused on the impact of a single component in the food environment; however, taking a whole systems approach resulted in more suitable food provision to meet the dietary needs of athletes.

## 1. Introduction

High-performance athletes regularly travel and compete at major competition events. Many of these events involve residing in a village environment where meals are catered during a 24 h period. There is a number of barriers to providing suitable food to meet the performance, cultural, and special dietary needs of athletes, despite evidence that nutrition is important to performance [1]. This is predominately driven by budgetary constraints and impacted by the local environment and location of the event [2]. A review of food provision at major events identified that contemporary issues such as environmental sustainability and food safety, particularly in light of COVID-19, are the main focus for caterers [3]. There is also evidence to show that athletes do not always eat suitable food while competing at these events [4], which may be related to not prioritising performance when choosing food [5] or lack of knowledge about eating for performance [6]. Furthermore, interventions designed to improve the nutrition knowledge of athletes are not always successful and have variable outcomes on changing dietary intake [7,8]. 

There has been recent debate as to whether a change in eating behaviours rests with the individual or sits with the food sector, particularly when eating outside of the home [9]. It has been recognised that change or redesign to the food environment has the potential to influence consumers’ food choices [10]. Strategies that encourage healthier food choice away from home use choice architecture to nudge consumers to choose particular foods [11]. The more commonly implemented strategies that promote behaviour change, such as nutrition labelling of menu items, educational information, and changes to serving plates and cutlery, have inconclusive outcomes [10]. Research in recreation and sports settings has suggested that multi-level setting-based approaches may be more impactful than a singular intervention [12]. Menu labelling alone has been shown to have a moderate impact on altering the intake of specific nutrients and dietary components of consumers [13], yet the impact on athletes’ diets is unknown. Nutrition labelling of menu items is commonly provided in the athletes’ dining hall at major events and has been identified as important by athletes [14] but is not always used when making food choices [4]. The provision of healthy food has also been identified as a way to create change with minimal effort from the consumer. This can be at the level of policy action through offering alternative food items or reformulating foods.

A systematic review of food service initiatives to help consumers make improved food choices [9] identified three types of initiatives or schemes: ‘informing’—requiring the consumer to interpret and understand information to make a food choice; ‘enabling’—a structural change resulting in making healthier choices easier; and ‘engineering’—which resulted in changes to individual foods, products, or menus so the consumer does not need to make a choice to eat healthier. The outcomes of this review suggested that the food service sector could take action to shape the food environment [9]. For athletes at major events, the provision of suitable food by caterers is vital since athletes’ food choices can impact their fuelling, recovery, weight control, and gut comfort, with implications for their sports performance. 

Strategies that impact the food environment have been shown to be successful in the provision of suitable food to athletes at a number of past competition events. These strategies included engineering (review of the menu by experts in advance of the event) [15,16], informing (nutrition labelling of items, website, resources [14,17,18,19]), and enabling (nutrition desk with expert consultations, tours of the dining hall, meal plans for athletes, staff training [18,20,21]) schemes. These have predominately been the result of the integration of nutrition experts working alongside the caterers [2]. However, input from nutrition experts has not been consistent, and thus, there have been varying outcomes in terms of the suitability of the food provided at major events [15]. This appears to be driven by the experience of the caterers, the location, and the early input of nutrition expertise [2]. There has also been an increasing expectation that the food will meet the specific and very individual dietary and performance needs of athletes while complying with the organising committee’s constraints and budget [2].

Since the Sydney 2000 Olympic Games [18], no studies have comprehensively evaluated the impact of a combination of strategies on the whole food environment. Recent research has identified the complexity of food provision in this environment and suggested that a nutrition program should be better integrated into the food service system model, which encompasses planning through operations [2], while still considering global challenges relevant to this environment. The aims of this study were to (1) map the range of food service nutrition schemes that were implemented prior to and during a major competition event (the 2018 Commonwealth Games) and (2) evaluate the process, impact, and short-term outcome of these schemes through staff training satisfaction, athlete feedback, nutrition service usage, and quality assurance checks. 

## 2. Materials and Methods

### 2.1. Setting

This study followed a case study design in a real-world setting with multiple interventions [22] and descriptive outcomes. The setting for this study was the 2018 Commonwealth Games, Gold Coast, Australia (18 sports and 7 para-sports, 6600 athletes and officials, and 71 countries) [23]. This event provided the opportunity to implement strategies that impacted the whole food environment, from planning to operation. The main focus for the intervention was the main dining hall of the athletes’ village, where athletes and teams ate their meals throughout the course of the competition event. The dining hall was a large-scale site that seated around 4000 individuals and provided food at multiple hot (staff-served) and cold (self-service) service areas during 24 h of operation. The dining hall is the primary location for teams to eat their meals during major competition events. 

### 2.2. Nutrition Program

The environmental nutrition intervention was implemented during both planning and operation, with specific goals, as outlined in Table 1. 

The preparation phase was from 10 January to 19 March 2018 and the operational phase from 19 March to 18 April 2018. The research was conducted in accordance with the Declaration of Helsinki, and ethical approval was granted by the Ethics Committee of the University of the Sunshine Coast (HREC no. 1/71/086). 

The environmental nutrition intervention schemes implemented prior to and during this event included:

1. Informing schemes
  - Development of nutrition labelling identifying nutrient and allergen content located at point of service (planning phase)
2. Enabling schemes
  - Training staff (catering management, service and floor staff—‘front of house’, chef and cooks—‘back of house’) prior to the event (planning phase)
  - Nutrition service provided by sports dietitians during the event (operational phase)
  - Gluten-free food station with toaster and food items (operational phase)
3. Engineering schemes
  - Review and modification of the menu and food service through expert recommendations and input (planning phase)
  - Attendance of a nutrition expert at catering management meetings onsite to request menu changes (operational phase)

### 2.3. Informing Scheme: Nutrition Labelling

Nutritional analysis and coding for allergens of individual menu items based on ingredient lists were conducted by the research team (dietitians) using dietary analysis software created specifically for this project. This then informed the nutrition labelling for each menu item. The label displayed ingredients, serving size, nutritional breakdown (energy (kJ/kcal), protein (g), total and saturated fat (g), carbohydrates and sugars (g), sodium (mg), and fibre (g)), and symbols representing special diets (vegan, vegetarian, Halal), allergens (gluten, pork, shellfish, fish, nuts, dairy, eggs) and other dietary needs (low sodium, low energy, spicy). 

### 2.4. Enabling Schemes: Staff Training, Nutrition Service, and Gluten-Free Food Station

Staff training sessions were held with all catering management and staff in advance of the event (February 2018) as part of a required induction to working at the event. The training sessions were presented by an Accredited Practising Dietitian and included topics of food safety, food allergens and cross-contamination, performance nutrition for athletes, and customer service. For the chefs, the training topics included information on the standardised recipes and athlete food choices (i.e., plain items, low fat, low sodium) instead of the customer service topic. 

A nutrition service was provided in the form of a nutrition desk located close to the entrance of the dining hall within the athletes’ village. The purpose was to field athlete and team enquiries about the food provision and menu, and for any particular requests, to provide a nutrition consultation service, to provide group education and guidance on menu choices, to be a point of contact for requests to take to catering staff, to monitor a gluten-free food station located near the desk, and to provide weight check-ins for athletes. The desk was staffed by four experienced sports and food service dietitians, and the desk was open for 12–17 h for the duration of the dining hall’s operational period (20 March to 18 April). All patrons within the dining hall had access to the service and could visit on request. 

A gluten-free station was implemented during the event based on recommendations by dietitians during the planning phase. The gluten-free station provided items such as gluten-free bread, biscuits, breakfast cereal, and baked items, as well as dedicated toasters. The items that were provided at the station were determined by the expert dietitians to not contain gluten as per the ingredient list and labelling requirements. The station was located near the nutrition desk and was monitored by the dietitians to reduce cross-contamination risk. 

### 2.5. Engineering Scheme: Nutrition Expert Integration into Catering

Review of the menu for cultural, performance, and special dietary needs was conducted by the researchers as experts in food provision at major events in the planning phase. Recommendations were provided in a report and through regular meetings with catering staff. All dietary analysis and coding of ingredients and recipes was conducted by the nutrition team (food service dietitians). Regular meetings with caterers were held during the planning and operational phases to ensure implementation of the recommendations. 

### 2.6. Evaluation Measures

This study involved complex program evaluation of the interventions above, which includes multiple methods to produce a more effective impact on a population group [24]. Evaluation is defined as reflexive intervention and measuring short-term impact and long-term outcome [24]. Process evaluation in this setting included staff feedback and satisfaction on the nutrition training. Impact evaluation included quality assurance checks of serving sizes of food by researchers, nutrition desk visits and feedback/queries, and nutrition labelling, and outcome evaluation was determined to be patron feedback on the menu. Ratings for the menu were compared against participants grouped based on how useful they found the nutrition labelling, nutrition staff, and serving staff in helping them to identify meals/items to meet their needs (‘useful’ or ‘very useful’ vs. ‘did not use’ to ‘somewhat useful’). Longer-term outcome evaluation beyond the life of the event was not undertaken. The relationships between the scheme, intervention, data collection, and evaluation are provided in Table 2. 

### 2.7. Process Evaluation: Staff Training Survey

As part of the process evaluation, on completion of the training presentation, all staff in attendance were invited to complete a paper-based evaluation survey. The survey included a series of questions about opinions on the length of the training, the relevance of their role to the training material, their interest in the material presented, their understanding of the material, their confidence in referring to a nutrition desk, and their overall satisfaction with the training (1–5 Likert scale). 

### 2.8. Impact Evaluation: Quality Assurance and Nutrition Service

Menu items were dished up by service staff, and nutrition card information about the items was recorded along with the weight (in g) and tested for taste and sensory appeal by eight trained members of the research team (expert testers). Menu items were randomly sampled across all four meal periods and from all hot service areas. Testing was conducted from 20 March to 16 April. Data were recorded in an online form at the time of testing. Data analysis included differences between the menu card serving size and the actual weighed amounts. 

Structured records of all enquires, consultations, education sessions, and weigh-ins were kept by dietitians at the nutrition desk for the duration of the event (20 March to 18 April). The dietitians also used their expert opinion on a scale of 1–5 (‘very poor’ to ‘very good’) to rate the nutrition knowledge and practice of each individual athlete that had a consultation as a means for reviewing the need for the service. The athletes were asked how confident they were in their nutrition knowledge on a scale of 1–5 (‘not at all confident’ to ‘very confident’). The athletes were also asked how well they put their nutrition knowledge into practice with response options of ‘Poor—I rarely follow the diet I know is right’, ‘Below average—I apply what I know only some of the time’, ‘Average—I apply what I know half of the time’, ‘Above average—I apply what I know most of the time’, and ‘Excellent—I apply what I know in practice nearly all of the time’. Records were kept on the usage of the gluten-free station based on sex, country, and role (athlete or not). 

### 2.9. Impact and Outcome Evaluation: Patron Dining Hall Survey

This study used convenience sampling to survey dining hall patrons for their feedback on multiple facets of the dining hall. The dining hall survey was available in hard copy at the nutrition desk located near the main entrance of the dining hall. All patrons (athletes, coaches, other team members) that passed by the desk had the opportunity to participate. After a brief conversation with participants to subjectively determine their English capabilities, the researchers sought verbal consent and offered the survey for completion. All participants were entered into a prize draw awarded upon completion of the event. Participants were free to stop completion and not return the survey if desired. All surveys were anonymous, and participants took 10–20 min to complete the questions. Sampling took place until the closing of the dining hall. 

The survey included questions about the nutrition labelling, food environment, staff knowledge, nutrition service, menu items, and provision for particular needs (e.g., gluten free). The questions were based on previous surveys developed to evaluate food provision in this environment [21,25]. Participants rated, on a 1–5 scale (‘very poor’ to ‘very good’), 13 attributes of the food provision and whether they thought there were sufficient menu items across 12 categories relevant to the provision of food to meet performance, special dietary, and cultural/religious needs. 

### 2.10. Data Analysis of Surveys

Data from the two surveys were entered into the online version via surveymonkey.com. Data analysis included the use of Microsoft Excel (2013, Microsoft Corporation, Redmond, WA, USA) and Statistical Package for Social Sciences (SPSS) Statistical Software (version 28.0, IBM Corporation, Armonk, NY, USA). 

For both surveys, responses to the Likert scale-type questions were tested with collapsed categories for consistency, as many variables contained small counts that violated test assumptions. Categorical data were examined via Chi-squared analysis, while the Mann–Whitney U and Kruskal–Wallis H tests were used to examine differences for continuous data. Significance was set at 0.05, and the Bonferroni correction was applied when examining post hoc results. Descriptive results are reported with the number (*n*) and median (Md) or proportion (%), and age is reported with the interquartile range (IQR). The sports were grouped into six categories based on previous Commonwealth Games research [26]. Open-ended responses were categorised into themes by one researcher and checked by the other, with any discrepancies resolved through discussion.

## 3. Results

The evaluation of all schemes was determined based on the following results:

- Responses to the dining hall survey;
- Data collected from the nutrition service and gluten-free station;
- Responses by staff to the training survey;
- Data collected from the quality assurance process.

### 3.1. Participant Characteristics: Dining Hall Survey

Patrons (*n* = 322) who were considered end users of the schemes completed the survey. This included impact evaluation for labelling (informing) and the nutrition service (enabling) and outcome evaluation for the staff training (enabling) and catering integration (engineering). The majority of respondents to the survey were athletes (*n* = 219, 68%) and females (*n* = 183, 57%). The female participants were younger than the males (Md = 26, IQR = 23–35 vs. Md = 30, IQR = 24–44, U = 9866.5, *p* = 0.005, *n* = 314). Demographic details are provided in Table 3. 

### 3.2. Informing Scheme: Nutrition Labelling

Ratings for the nutrition cards used to display nutrition information about the menu were high, with 80–88% of participants rating all five card attributes as ‘good/very good’. The serving size information was the lowest-rated attribute, receiving ‘average’ or ‘low/very low’ ratings by 17% and 3% of respondents, respectively. Comparatively, the ratings for the dietary needs, presentation, nutrient content, and ingredient attributes received rating responses of ‘average’ by 10–13% and ‘low/very low’ by 0.7–1.4% of participants. 

### 3.3. Engineering Scheme: Catering Engagement during Planning and Operation

#### 3.3.1. Menu Rating

**Figure 1:**
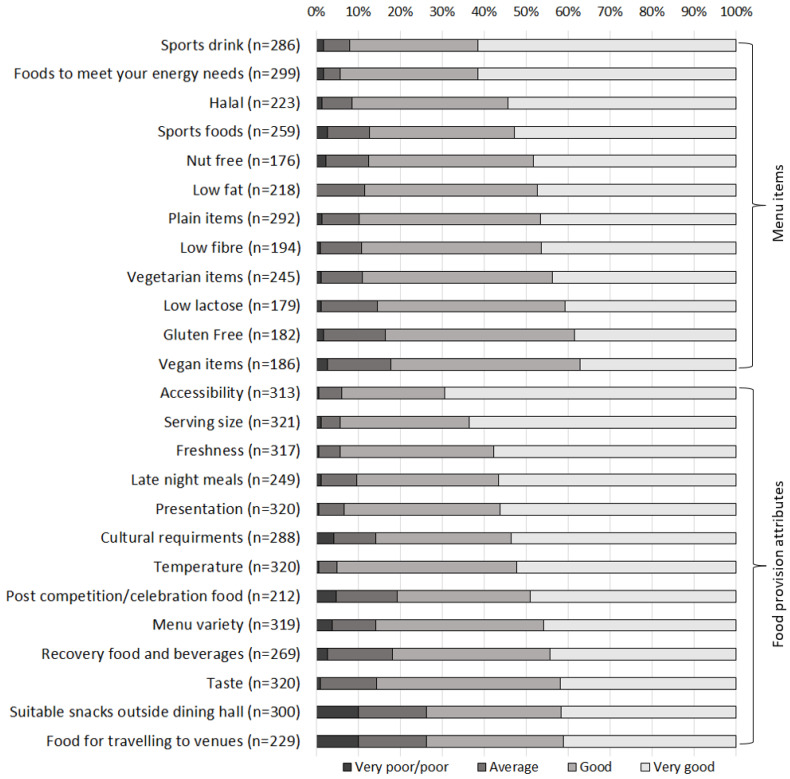
Proportion of ratings by dining hall respondents (*n* = 323) for food provision and specific menu items.

The overall rating of the menu by patrons was a mean of 8.6 ± 1.4 out of 10. Athletes rated the food and beverages higher than other delegates (Md = 9 vs. Md = 8, U = 9866.5, *p* = 0.044, *n* = 323). All categories received high proportions of ‘good/very good’ ratings (82–94%). The highest-rated item categories were for there being ‘enough foods to meet your energy needs’, followed by there being ‘enough sports drinks’. The lowest-rated item types were for there being enough vegan items, followed by enough gluten-free items (Figure 1). Males considered the provision of low-fat items as better than females (Md = 5; ‘very good’, CI = 5–5 vs. Md = 4; ‘good’, CI = 4–5; U = 4803.0, *p* = 0.027, *n* = 217). Athlete participants in aesthetic/weight-category sports rated the provision of sports foods better than their racket sport counterparts (Md = 5; ‘very good’, CI = 5–5 vs. Md = 4; ‘good’, CI = 4–5; H = 14.6, df = 5, *p* = 0.012, *n* = 179). 

#### 3.3.2. Ease of Finding Items

Overall, the participants found it easy to find items on the menu that met their nutritional/dietary needs (Md = 4; ‘good’, range 2–5; ‘poor’–‘very good’). No significant differences were detected across participant characteristics. Athletes who felt it was ‘easy/very easy’ to find items on the menu that met their needs were significantly more likely to report various menu item categories as being ‘good/very good’, including plain (*p* < 0.001), low-fat, (*p* = 0.003), and low-fibre items (*p* = 0.006), as well as sports foods (*p* < 0.001) and foods to meet energy needs (*p* < 0.001). 

#### 3.3.3. Attributes of the Food and Beverage Provision

Attributes of the menu received ‘good/very good’ ratings by 74–95% of participants (Figure 1). The lowest-rated attributes were the provision of suitable snacks for taking out of the dining hall and the provision of food for travelling to venues, both of which received ‘average’ to ‘poor/very poor’ ratings (*n* = 79, 26% and *n* = 60, 26%, respectively). Those from Western regions rated taste higher than those from African regions (Md = 5; ‘very good’ versus Md = 4; ‘good’; H = 8.2, df = 3, *p* = 0.042, *n* = 310), as well as cultural requirements (Md = 5; ‘very good’ vs. Md = 4; ‘good’; H = 23.8, df = 3, *p* < 0.001, *n* = 278) and provision of food for travelling to venues (Md = 4; ‘good’ vs. Md = 4; ‘good’; H = 9.9, df = 3, *p* = 0.019, *n* = 220). 

A total of 96 comments were received about the menu. Some comments were generic positive statements, such as, “I like the food so much,” “good service—deserve to be congratulated,” “the food is lovely,” and “love the GF toaster and range away from the rest.” Other comments focused on specific areas for improvement. The majority were related to cultural requirements (e.g., “some cultural food is not real culture food. African tastes like Asian”), snacks (e.g., “need more snacks to go such as baguettes, pasta bowls, sandwiches”) and beverages (e.g., “more flavoured milk”). 

### 3.4. Enabling Scheme: Nutrition Service

The nutrition desk provided a total of 4064 weight checks, 214 enquires, 62 consultations, and 25 requests for meals in isolation. Characteristics of athletes and delegates utilising nutrition services, along with example enquiries received, are outlined in Table 4. A number of individuals approached the nutrition desk about their food allergies and intolerances and their dietary requests. This included gluten free, soy allergy, nut allergies, lactose intolerance, vegan, MSG, and vegetarian (no eggs). In addition, there were requests for specific food such as Ugali, tofu, raw eggs, and cooked salmon fillets. 

Consultations were most commonly undertaken with athletes (Table 4), with the majority being with those in weight-category sports (*n* = 10 (boxing, weightlifting)), power sprint (*n* = 9 (track and field events)), and endurance sports (*n* = 9 (cycling, swimming, triathlon)). Consultations with boxers and weightlifters were generally in relation to making weight for competition, with athletes varying from 1 to 6 kg over their designated weight category. Only five athletes reported having a competition nutrition plan. When asked how confident (1–5 scale) they were in their nutrition knowledge, 37 of 42 (88%) athletes responded as ‘not feeling confident at all’ (*n* = 9, 24%) or ‘only a little confident’ (*n* = 20, 54%). The athletes (*n* = 36) rated how well they put their nutrition knowledge into practice, ranging from ‘poor’ (rarely following the diet they know is right) to ‘excellent’ (applying what they know in practice nearly all of the time). The majority of athletes (*n* = 20, 54%) felt they were average in applying their nutrition knowledge in practice. The dietitians’ ratings for nutrition knowledge and dietary intake (1–5 scale) were recorded for 37 athletes. Nutrition knowledge was rated mostly as ‘poor’ (*n* = 15, 41%), followed by ‘average’ (*n* = 13, 35%), whereas dietary intake was rated mostly as ‘average’ (*n* = 20, 54%), followed by ‘poor’ (*n* = 9, 24%). Seventeen comments were received by athletes on the ease or difficulty of following their nutrition plan in the dining hall, with the most common comment (*n* = 10) related to the number of choices and amount of temptation. 

### 3.5. Enabling Scheme: Gluten-Free Station Utilisation

A total of 88 individuals (64% athletes and 80% female) registered at the gluten-free station as regular users. The majority of users were from England (19%), Australia (17%), South Africa (13%), and New Zealand (11%). 

### 3.6. Impact and Relationship of Schemes: Usefulness of Nutrition Cards, Nutrition Service, and Serving Staff

When asked how useful the schemes were in helping to identify meals/items to meet their needs, the majority of participants found the nutrition cards (*n* = 227, 71%) and serving staff (*n* = 212, 66%) ‘useful/very useful’ for finding items on the menu. A smaller proportion of participants reported not using the nutrition card or serving staff (21 and 22%, respectively) compared to those not using their teammates or coach (35%) or the nutrition staff (40%). Although there was higher use of the serving staff, they received more “not useful” responses (6%) than the nutrition staff (3.5%) and nutrition cards (2%). Further differences in ratings of usefulness were detected between participants based on their sex, region, and sport (Table 5a). 

The number of schemes that were rated as ‘useful’ or ‘very useful’ by participants (*n* = 313) ranged from none (20%, *n* = 63) to all three schemes (41%, *n* = 133). The proportion that rated one or two schemes as ‘useful/very useful’ were 14% *n* = 45 and 25% *n* = 82, respectively. Participants from Western regions were less likely to rate all three schemes as ‘useful/very useful’ (20%, *n* = 26) compared to participants from the Africa (38%, *n* = 49) and Asia/Pacific regions (27%, *n* = 34; X^2^ = 25.5, df = 9, *p* = 0.004). 

Participants (*n* = 316) who rated the nutrition staff as ‘useful/very useful’ gave a higher median rating for the menu (Md = 9, CI = 9–10) compared to those who either did not use them or rated them as having ‘not useful’ to ‘average’ usefulness (both Md = 8, CI = 8–9; H = 31.1, df = 2, *p* < 0.001). There was no relationship between menu rating and serving staff and labelling. Ratings for the usefulness of serving and nutrition staff were examined against athlete ratings for the two lowest-rated attributes of the food and beverage provision (Table 5b). Overall, athletes were more likely to rate the provision of suitable snacks for taking out of the dining hall and the provision of food for travelling to venues as ‘good/very good’ if they also found the nutrition and serving staff to be ‘useful/very useful’. No significant differences were detected for the usefulness of the nutrition card information. 

### 3.7. Results from the Enabling Scheme: Staff Training

#### 3.7.1. Process Evaluation of Staff Training

The staff training survey received 310 responses; 39% (*n* = 120) stated that this was a new role and 76% had no previous nutrition training (Table 6). The role of the respondents varied and included managers, sous chefs, chefs, kitchen stewards, runners, front-of-house service staff, team leaders, cleaners, baristas, and muster room and other events support staff. Staff that were 30 years of age or older were more likely to have received prior nutrition training (*n* = 36, 31.6%, *p* = 0.019) and have more than 2 years of experience in their role (*n* = 60, 52.6%, *p* < 0.001; Fisher’s exact, *n* = 310). 

The nutrition training was well received, with most participants (*n* = 200, 66%) being ‘very satisfied’ or ‘somewhat satisfied’ (*n* = 49, 16%). Overall, 57% (*n* = 174) of respondents ended the training feeling ‘very confident’ in being able to refer to the nutrition desk, followed by 39% (*n* = 119) feeling ‘somewhat confident’ and 3% (*n* = 8) ‘not confident’. Between 87 and 90% of respondents stated that they ‘agree/strongly agree’ that the training helped with their understanding of nutrition labelling, food allergy and intolerance, and nutrition for athletes. A similar proportion (82–88%) of positive responses was received for respondents that “agree/strongly agree” that the information was relevant to their role; they were interested in the presentation and considered the presenter to be engaging and that they had learnt something new. Comments (*n* = 67) were generally positive (e.g., “very interesting”, “very informative”, “visual aids that help to understand what was said”); however, some comments were received about the time for training (e.g., “we need more time for training”, “make the speech short”, “better screen”). There were no significant differences in responses across participant characteristics. 

#### 3.7.2. Impact Evaluation of Staff Training: Quality Assurance of Menu Items

The serving size weight on the nutrition cards was 9.4 g less for a sample of 245 items than the actual serving weight. Only 28% of cards displayed a weight within 10% of the actual weight of the item (Table 7). Items were tested from one to six times. Of those tested four or more times, the greatest average discrepancy above the serving weight listed on the card was lentil dahl (24 g more) and below the serving weight on the card was spinach and ricotta cannelloni (111.5 g less). There was a large variation in test weight for vegetables, rice, pasta, and noodles. 

A total of 739 comments (372 positive, 131 neutral, 236 negative) were received by expert testers in relation to the sensory properties of the item (*n* = 560), the food preparation (*n* = 165), and the food service (*n* = 14). Positive comments were mostly for the taste, appearance, texture, and cooking technique. Negative comments focused on specific issues related to the service (e.g., “shepherds pie had no mince plated”, “poor staff knowledge on how much to serve”) and the food’s sitting time (e.g., “food needing to be stirred”, “sauce splitting”). There were 424 comments about the nutrition information card across the following components: title, serving size, ingredients, energy and macronutrients, dietary symbols, and sodium. The majority (*n* = 377, 89%) indicated that they displayed incorrect information compared to what was served. This was in relation to the ingredients (*n* = 124, 33%), serving size (*n* = 115, 31%), and energy and macronutrient content (*n* = 51, 14%). Additional comments on the menu items are provided as Supplementary Materials Table S1.

#### 3.7.3. Outcome Evaluation of Staff Training: Dining Hall Survey

A majority (>90%) of ‘good/very good’ ratings were received from respondents for the speed of service, staff politeness, staff knowledge of the menu, and the tidiness and cleanliness of the dining hall. There were no significant differences based on demographic characteristics.

## 4. Discussion

This study aimed to map and evaluate the various nutrition intervention schemes implemented at the 2018 Commonwealth Games during the planning and operational phases of the event. The process, impact, and outcome evaluations, including a range of interventions of informing (nutrition labelling), enabling (staff training and nutrition labelling), and engineering (expert input into catering) schemes, demonstrated that the complex nutrition program was overall successful and that multiple approaches in both planning and operation are needed to provide food suitable for athletes at major competition events. 

Process evaluation of staff training demonstrated that the training was well received by the participants, although the attendees were generally well educated, had had previous nutrition training, and were skilled in hospitality. During planning, workforce training is important to ensure that catering staff understand the relevance and importance their role has in providing suitable food to athletes. The impact of staff training was evaluated through quality assurance testing of menu items and compliance with nutrition labelling across the operational phase. This showed variability across the tested menu items, predominantly in the ingredients, serving size, and energy and macronutrient content of the items. Comments by the testers reflected the discrepancies (for example, “the food was too oily”). This suggests poor compliance by chefs and cooks with standardised recipes, particularly around the use of oils/fats and seasonings and inaccurate standard services by front-of-house staff in hot service areas. Despite these results, outcome evaluation of this scheme via the dining hall survey suggested that patrons were overall satisfied with the food provided in the dining hall. The overall mean rating of the menu was high (8.6 ± 1.4 out of 10), which compares favourably to other events with similar feedback (7.8 ± 1.5; *n* = 390 from an unpublished report by authors, Taipei 2017 Universiade) as well as overall ratings of 5.0 and 8.0 by expert dietitians attending the 2012 and 2016 Olympic Games, respectively [15,16]. Patrons also agreed that the staff had good knowledge of the menu, were polite, kept a clean and tidy dining hall, and were useful in helping with snacks to take out of the dining hall as well as assisting with availability of food items to take to venues. 

Nutrition labels as an informing scheme have been suggested to be ineffective when used on their own to educate and promote informed choices [7,17]. A systematic review on interventions to promote healthier meals [27] concluded that nutrition labelling only impacted a small proportion of people and was not an effective strategy in changing food purchasing (or, in this case, food selection). Regardless, athletes use labels and have an expectation that they be provided [14]. In this study, it appeared that those using the nutrition labelling also used other schemes, such as the nutrition desk and serving staff, to assist them (*n* = 133, 41% of participants rated the three schemes as ‘useful/very useful’). It was also apparent that athletes from Western countries were less likely to use any of the supports/schemes to guide their food choices. In particular, a high proportion of females from Western countries did not use the nutrition staff. The use of labelling and other enabling schemes such as the nutrition desk have previously been shown to be used by a higher proportion of athletes from non-Western regions [20,21]. In addition, athletes from these regions appear to be more influenced by their coach and teammates in making food choices and may be less likely to have a pre-competition meal plan or access to nutrition expertise on site [26]. Despite this, it is apparent from previous research in this environment that a proportion of athletes, regardless of region, could benefit from dietary improvement in the competition environment [4], and thus, enabling and engineering schemes may be of broader benefit across the entire population. 

Nutrition labels have been highly variable across different major events. The lack of a consistent approach means that staff and attendees may find understanding labelling difficult, as labels are not familiar to those working or attending multiple events. In this study, the majority of participants found the labels useful; however, this did vary across regions, with 39.6% from Western countries finding them less useful or not using them. Future labelling may focus on only essential information displayed at the point of choice, with more detailed nutrition analysis and non-critical allergens available electronically via a QR code scan. Examination of athlete understanding of labels would also be beneficial, as could exploring the components of the label that were most useful (for example, nutrition composition versus symbols for allergens). Research investigating implementation of menu labelling in food service more broadly suggests that a lack of standardised recipes, menu changes, and a fast-paced environment may create barriers to successful labelling and that the perspective of consumers (in this case, athletes) is important [28]. In this intervention, menu labels were printed and updated as required. Paper labels that constantly need updating as menu items change are not environmentally sustainable. Anecdotally, the nutrition team found it hard to keep up with labelling changes, as catering staff adapted the menu to suit the available food supply. This is a common occurrence and the reason why the use of technology for electronic labelling has been suggested as a means of adapting quickly to the changing environment [2].

For this event, a website was designed and planned for individuals to access detailed nutrition information in advance and during the event, but this did not receive approval by the organising committee in time to be implemented. Major events that have occurred during and post COVID-19 have implemented electronic communication about the menu, but this is of little use if not dynamically updated based on catering changes. Furthermore, testing of serve sizes demonstrated that paper labels were not reflective of the actual serving. We recommend that labels provide the name, key ingredients and allergens, and special dietary information at the point of choice, with nutrition information and a detailed ingredient list available through a QR code. Serve sizes should be listed per standard weight (100 g), as well as a “typical” serving with the descriptor of the serving utensil (for example, one ladle = 150 g). 

The nutrition desk was seen as a positive supportive service for athletes, especially with a visible location near the dining hall’s entrance. The use of scales for weight checks at the nutrition desk attracted athletes mainly from weight class sports and allowed for casual interaction about their dietary intake, with many returning for further consultation. We found that those who used the nutrition staff and found them useful rated the menu higher overall and their specific needs for food to take out of the dining hall and for travelling to venues also more highly. Both of these situations have been poorly rated by individuals at past events [15,16], yet these events did not provide the same level of nutrition service in the dining hall as the current study. This suggests that a prominent nutrition service with expert staff can help to problem solve and provide solutions for teams and athletes at front of house and provide a link catering that ultimately assists with appropriate food provision. The results of this study suggest that there is a clear need for a nutrition service, particularly given that the experts’ ratings of dietary intake and the nutrition knowledge of those receiving consultations were generally ‘average–poor’. There is evidence to suggest that nutrition interventions conducted over a brief time period that include some education, such as the nutrition service provided at this event, can impact short-term nutrition outcomes [29]. Thus, in theory, the impact of this service would extend beyond the life of the competition event, although evidence is lacking. Despite this, not all patrons make use of the nutrition service, hence the importance of including engineering schemes for broader reach.

The 2018 Commonwealth Games occurred in a location where procurement of food was not an issue (Gold Coast, Australia), although it was held during a public holiday period, resulting in some issues with the supply chain. Anecdotally, at the 2017 Universiade Games in Taiwan, where the supply of gluten-free foods was limited, the nutrition team sourced items from outside the country. At the current event, access to gluten-free items was not restricted, and a gluten-free station was situated near the nutrition desk to enable changed behaviour by athletes who needed this service. Athletes and teams that attend successful events such as the 2018 Commonwealth Games develop an expectation around excellence in food provision and have an expectation of similar standards from one to the next [2]. There is a question as to whether caterers can keep up with the increasing demands of teams. More recently, some countries have provided their own supplementary food at events, but this has predominately been the result of COVID-19’s impact on food service delivery [30]. 

Although it is difficult to assess how the nutrition service ultimately impacted or changed athletes’ food choices, this scheme required more interactive engagement from the caterers and less decision-making by the athletes who used the service. Previous studies investigated athletes’ self-reported influences on food choices [5] and actual food selection [4]; however, this has not been directly linked to those using the nutrition service. Future studies that link the use of enabling and informing schemes to food choice and dietary intake would provide more robust outcome evaluation. The input of nutrition expertise during the planning phase has been variable at past events [3] yet can be a valuable engineering scheme with a broader impact on food provision. The current outcome evaluation of menu ratings by patrons may be due to experienced caterers and a nutrition team with a previous understanding of catering for major competition events. 

In the environment of a major event, it is difficult to measure the outcome evaluation of engineering schemes. The menu needs to cater to many cultures and sporting requirements, and athletes still need to be directed through enabling schemes to eat appropriately. Hence, patrons’ opinions of the menu is of value as an outcome measure of the suitability of the food to meet their specific sporting and cultural needs. In studies that focus on health, outcome evaluation can be measured through individual health outcomes. In athletes, the equivalent would be sports performance, but given that there are many physical and psychological factors that impact performance, this may not be directly related to food provision. Further long-term outcomes could be investigated by comparing similar schemes from one event to the next. 

A strength of this study is that it looked at a whole-system approach to achieve suitable food provision for athletes at this event, which has not commonly been reported in the literature [27]. Past major events that have been less successful in terms of food provision have focused on a single component of change to the food service (such as a nutrition service or labelling). Literature from other settings suggests that, despite informing schemes empowering individuals to make their own choices, they have limited impact when used in isolation [9,27], particularly in environments where there is time pressure with little conscious effort [31,32]. Enabling and engineering schemes require less conscious effort and can reach a broader number of athletes, including those who are less knowledgeable and engaged with nutrition for health and performance. 

Four guiding principles (availability, pricing and placement, promotion, and provider commitment) have been recommended for successful implementation of healthy food environments in recreation and sports settings [12]. As demonstrated through the results of this study, these can be adapted and applied in the context of major competitions. Implementation of these strategies can be supported through the integration of nutrition expertise during the menu and labelling design and staff training phase and a nutrition service by sports dietitians supporting appropriate food choices on site during operation. With changes in food provision post COVID-19 and the ongoing concerns about athlete safety [30,33] and security [3], as well as increased focus by athletes on appropriate nutrition in line with self-prescribed [2] and evidence-based dietary trends [34,35] and the focus on sustainability at major events [3,36,37], more action by food service providers will be needed. In agreement with literature in the broader health context [9], we recommend the creation of a policy framework for food service delivery in this environment. 

Furthermore, future interventions should consider applying the same multi-schemed approach with a sustainability framework [38] to determine whether food provision that is suitable for athletic health and performance addresses issues such as plate and bulk waste, procurement of local and seasonal food, and efficient production. For example, a novel engineering scheme could change meat and animal ingredients with plant-based ingredients, which has been suggested as an effective way to both reduce the environmental impact and improve dietary intake [39]. Additional research should also consider the specific needs of more diverse athletic groups, including Paralympic athletes who have specialised dietary needs [40]. 

### Limitations

Interventions of this nature are difficult to control, but taking advantage of the natural environment is important for evaluating environmental strategies [41]. Use of both objective and perceived measures can provide a more comprehensive picture of the impact of the strategies. This can also guide whether the implementation of the strategies was a success. We note that there will be variability in the delivery of the intervention schemes depending on the location. For example, training of staff may be more challenging in locations where there are higher numbers of unskilled or younger workers or where the language differs from those conducting the training. As the respondents to the surveys were a sample of convenience, sampling bias may have occurred. However, the surveys were collected next to the main entrance to the dining hall and incentive prizes resulted in broad representation across regions and sports. The environmental intervention was specifically targeted to the main dining hall, although all schemes would have had broader impact across the other smaller food outlets in the athletes’ village. Food provided at competition venues was provided by other caterers and outside the scope of this intervention. 

## 5. Conclusions and Future Direction

This study has demonstrated that a nutrition intervention that encompasses the entire food environment at major competition events can ultimately impact athletes’ ability to choose appropriate food for performance. The mapping of the informing, enabling, and engineering schemes and the results of the process, impact, and outcome evaluations implemented in this setting demonstrated positive outcomes. In particular, the integration of nutrition expertise in catering (engineering schemes) showed added value in impacting patrons’ opinions of the menu and food service. 

Increasing pressure on caterers to comply with budgetary constraints, sponsorship agreements, and sustainability efforts while providing safe food in a safe environment consolidates the need for a consistent and unified approach between events. The systems model proposed for delivery of catering for major sporting events [2] has identified that the ultimate change for a consistent approach is at the level of policy and tender for catering budget. As per the literature from the broader health context and the positive outcomes in terms of dining satisfaction from this comprehensive study, we recommend the creation of a policy framework that incorporates a variety of schemes that allow for safer and more appropriate food provision at major sporting events. 

## Figures and Tables

**Table 1:** Objectives of the nutrition program.

Planning and Preparation
1. To ensure that the menu meets the dietary, allergen, nutrition, and performance needs of all athletes and officials through expert review by experienced sports dietitians
Operational Phase
1. To provide a service offering sports nutrition education, advice, and counselling to teams, individual athletes, coaches, and officials who may have limited access to sports dietitians in their home country
2. To promote the importance of optimal nutrition at elite-level competition and the value of expert nutrition advice as it relates to overall health and sporting performance
3. To provide sports nutrition advice for athletes relevant to training and competition performance, including recovery, hydration, and weight management
4. To provide support to athletes with special dietary requirements for medical needs/illnesses pertinent to their health and wellbeing (coeliac disease, diabetes, food allergies/intolerances), religious beliefs (Halal), and personal preferences (vegan, vegetarian).
5. To provide nutrition guidance and expert advice for athletes, coaches, and officials to make informed choices while eating in the main dining hall
6. To provide a conduit between the organising committee, catering, and patrons regarding queries and concerns about the menu and food provided
7. To conduct quality management and research activities and report back on patron experience of the menu, dining hall, and nutrition service to catering and organisers
8. To ensure that correct information about the menu and individual items is displayed and updated as needed

**Table 2:** Evaluation measures of the intervention schemes.

Scheme	Intervention	Data Collected	Who	Variables or Dataset	Process	Impact	Outcome
Informing	Nutrition labelling	Dining hall survey	All patrons	Labelling feedback/usefulness		x	
Enabling	Staff training	Staff survey	All staff	Satisfaction	x		
				Usefulness	x		
		Quality assurance	Menu items	Serve size accuracy		x	
				Food qualities—expert comments		x	
		Dining hall survey	All patrons	Staff menu knowledge, speed of service, staff politeness, dining hall tidiness and cleanliness			x
	Nutrition service	Nutrition desk: enquiry	All patrons	No. and type		x	
		Nutrition desk: consult and expert ranking of diet	Athletes Experts	No. and type		x	
		Weigh-ins	Athletes	No. over time		x	
		Dining hall survey	All patrons	Usefulness of both serving staff and nutrition staff		x	
	Gluten-free service area	Usage	Athletes	No. over time		x	
Engineering	Expert engagement with catering during planning and operation—change to menu and service	Dining hall survey	All patrons	Food environment, menu, provision for specific needs			x

**Table 3:** Characteristics of participants who completed the dining hall survey (*n* = 322).

	Total		Male		Female		
	*n*	%	*n*	%	*n*	%	*p*
Sex	322		139	43.2	183	56.8	
Age							0.008
29 years or younger	181	57.6	67	37.0 ^a^	114	63.0	
30 years or older	133	42.4	70	52.6	63	47.4 ^a^	
Region							NS
Africa	105	32.6	45	42.9	60	57.1	
Latin America and Caribbean	42	13.0	21	50.0	21	50.0	
Central and Eastern Europe	9	2.8	6	66.7	3	33.3	
Western ^^^	106	32.9	39	36.8	67	63.2	
Asia Pacific	60	18.6	28	46.7	32	53.3	
Role							0.004
Athlete	218	67.7	82	37.6 ^b^	136	62.4	
Delegate ^$^	104	32.3	57	54.8	47	45.2 ^b^	
Sport group (athletes only)							0.013
Aesthetic and weight category	59	27.3	22	37.3	37	62.7	
Endurance	32	14.8	17	53.1 ^c^	15	46.9	
Power/sprint	47	21.8	20	42.6 ^c^	27	57.4	
Racket	28	13.0	12	42.9	16	57.1	
Skill	18	8.3	7	38.9	11	61.1	
Team	32	14.8	4	12.5	28	87.5 ^c^	

Missing values for sex (*n* = 1), age (*n* = 9), and region (*n* = 1). NS = not significant. ^^^ Western includes Australia, New Zealand, Canada, the United States, and the United Kingdom. ^$^ Delegates include coaches (*n* = 32), medical staff (*n* = 24), officials, and unspecified delegates (*n* = 48). ^a^ Fisher’s exact text, difference between sex and age. ^b^ Fisher’s exact test, difference between sex and role. ^c^ Chi-squared test statistic X^2^(5) = 12.72; significant difference in the proportion of females in team sports in comparison to males in endurance and power/skill sports after Bonferroni correction (*p* < 0.05).

**Table 4:** Characteristics of participants who utilised the nutrition desk services and examples of the enquiries received.

	Enquiries	Consultations	Weight Checks	Enquiries	
	*n* = 214	*n* = 62	*n* = 4064		
Sex *n* (%)				Theme	Examples
Male	109 (51)	40 (65)	2734 (67)		
Female	104 (49)	22 (35)	1320 (33)	Sports nutrition	“What should I eat between weigh-in and competition?”
Role ^a^ *n* (%)					“Are these scales correct? They are 600 g lower than gym scales.”
Athlete	106 (51)	42 (70)	3469 (86)		“What supplements are best to take?”
Delegate	103 (49)	18 (30)	588 (14)		
Sport group *n* (%)				Menu/food service	“Where can I order or get food for shooting venue?”
Aesthetic/weight category	26 (33)	10 (24)	698 (20)		“When is the lactose free milk coming back in?”
Endurance	12 (15)	9 (21)	374 (11)		“Who do I talk to about takeaway food for at the games events?”
Power/sprint	21 (27)	9 (21)	1449 (42)		
Racket	1 (1)	6 (14)	178 (5)	General nutrition advice	“Can I get a meal plan for losing weight?”
Skill	6 (8)	2 (5)	295 (9)		“Is there a special diet for hypertension?”
Team	12 (15)	6 (14)	442 (13)		
TOTAL (number of sports)	18	12	34	Allergy/intolerance	“What type of bread would be the best low FODMAP choice?”
Region ^b^ *n* (%)					
Africa	50 (24)	16 (26)	1640 (41)	Other	“What do you do at the desk? I am a dietitian new grad and athlete.”
Latin America/Caribbean	21 (10)	26 (43)	273 (7)		“Where can I get ice for an eski from?”
Central and Eastern Europe	2 (1)	1 (2)	84 (2)		
Western	95 (46)	7 (12)	912 (23)		
Asia Pacific	37 (18)	11 (18)	1133 (28)		
TOTAL (number of countries)	46	26	66		

Missing values for sex = enquiry (*n* = 1) and weight checks (*n* = 10); for role = enquiries (*n* = 5), consultations (*n* = 2), and weight checks (*n* = 7); for sport group = enquiries (*n* = 28) and weight checks (*n* = 33); and for region = enquiries (*n* = 9), consultations (*n* = 1), and weight checks (*n* = 22). ^a^ Delegates include coaches, medical staff, officials, and unspecified delegates. ^b^ Western includes Australia, New Zealand, Canada, the United States, and the United Kingdom.

**Table 5:** (**a**) Ratings according to participant characteristics for the use of and usefulness of the nutrition card information, serving staff and nutrition staff. (**b**) Ratings according to athlete characteristics, ability to take snacks out of the dining hall, and food for travelling, in comparison to the use of and usefulness of the nutrition card information, serving staff and nutrition staff.

**(a)**																					
	**Nutrition Card Information**							**Serving Staff**							**Nutrition Staff**						
**All Participants (*n* = 320)**	**Did Not Use**		**Not Useful to Average**		**Useful or Very** **Useful**		***p***	**Did Not Use**		**Not Useful to Average**		**Useful or Very** **Useful**		***p***	**Did Not Use**		**Not Useful to Average**		**Useful or Very** **Useful**		***p***
	***n***	**%**	***n***	**%**	***n***	**%**		***n***	**%**	***n***	**%**	***n***	**%**		***n***	**%**	***n***	**%**	***n***	**%**	
Sex							NS							NS							0.016
Male	26	18.8	9	6.5	103	74.6		27	19.6	14	10.1	97	70.3		42	30.7	11	8.0	84	61.3 ^a^	
Female	42	23.2	16	8.8	123	68.0		43	23.8	24	13.3	114	63.0		83	46.6 ^a^	12	6.7	83	46.6	
Role							NS							NS							NS
Athlete	43	19.8	15	6.9	159	73.3		45	20.8	28	13.0	143	66.2		81	37.5	16	7.4	119	55.1	
Delegate	25	24.3	10	9.7	68	66.0		25	24.0	10	9.6	69	66.3		44	44.0	7	7.0	49	49.0	
Region ^^^							0.027							<0.001							<0.001
Africa	17	16.3	8	7.7	79	76.0		12	11.7 ^c^	16	15.5	75	72.8		31	30.7 ^d^	11	10.9	59	58.4	
Asia and Pacific	6	10.3 ^b^	5	8.6	47	81.0		7	11.9 ^c^	6	10.2	46	78.0		12	21.1 ^d^	5	8.8	40	70.2	
Western	32	30.2	10	9.4	64	60.4 ^b^		38	35.8	12	11.3	56	52.8 ^c^		63	59.4	6	5.7	37	34.9 ^d^	
Latin America/Caribbean	12	28.6	1	2.4	29	69.0		11	26.2	3	7.1	28	66.7		15	35.7	0	0.0	27	64.3	
Total	68	21.3	25	7.8	226	70.9		70	21.9	38	11.9	212	66.3		125	39.6	23	7.3	168	53.2	
**(b)**																					
	**Nutrition Card Information**							**Serving Staff**							**Nutrition Staff**						
**Athlete only (*n* = 217)**	**Did Not Use**		**Not Useful to Average**		**Useful or Very** **Useful**		***p***	**Did Not Use**		**Not Useful to Average**		**Useful or Very** **Useful**		***p***	**Did Not Use**		**Not Useful to Average**		**Useful or Very** **Useful**		***p***
	***n***	**%**	***n***	**%**	***n***	**%**		***n***	**%**	***n***	**%**	***n***	**%**		***n***	**%**	***n***	**%**	***n***	**%**	
Sex							NS							NS							NS
Male	15	18.5	6	7.4	60	74.1		17	21.0	9	11.1	55	67.9		23	28.4	7	8.6	51	63.0	
Female	28	20.7	9	6.7	98	72.6		28	20.9	19	14.2	87	64.9		58	43.3	9	6.7	67	50.0	
Sport ^^^							NS							NS							NS
Aesthetic/weight category	19	32.8	3	5.2	36	62.1		14	25.0	8	14.3	34	60.7		25	44.6	3	5.4	28	50.0	
Endurance	3	9.4	1	3.1	28	87.5		6	18.8	2	6.3	24	75.0		12	37.5	1	3.1	19	59.4	
Power/sprint	4	8.3	3	6.3	41	85.4		7	14.6	7	14.6	34	70.8		11	22.9	3	6.3	34	70.8	
Racket	5	18.5	3	11.1	19	70.4		4	14.3	6	21.4	18	64.3		13	46.4	5	17.9	10	35.7	
Team	4	22.2	2	11.1	12	66.7		5	27.8	2	11.1	11	61.1		8	44.4	1	5.6	9	50.0	
Region ^^^							NS							.006							<0.001
Africa	13	18.6	5	7.1	52	74.3		10	14.5	12	17.4	47	68.1		22	31.9	7	10.1	40	58.0	
Asia and Pacific	3	7.7	5	12.8	31	79.5		5	12.8	6	15.4	28	71.8 ^e^		7	17.9	5	12.8	27	69.2 ^f^	
Western	22	28.2	5	6.4	51	65.4		27	34.6 ^e^	10	12.8	41	52.6		46	59.0 ^f^	4	5.1	28	35.9	
Latin America/Caribbean	5	21.7	0	0.0	18	78.3		3	13.0	0	0.0	20	87.0 ^e^		4	17.4	0	0.0	19	82.6 ^f^	
Taking suitable snacks out of the dining hall							NS							.012							<0.001
Very poor to average	15	26.8	5	8.9	36	64.3		17	29.8 ^g^	11	19.3	29	50.9		34	59.6 ^h^	7	12.3	16	28.1	
Good to very good	25	16.4	10	6.6	117	77.0		25	16.7	16	10.7	109	72.7 ^g^		44	29.3	8	5.3	98	65.3 ^h^	
Provision of food for travelling to venues							NS							NS							0.009
Very poor to average	9	23.7	4	10.5	25	65.8		10	25.6	8	20.5	21	53.8		20	51.3 ^i^	3	7.7	16	41.0	
Good to very good	22	17.9	11	8.9	90	73.2		18	14.8	13	10.7	91	74.6		31	25.4	10	8.2	81	66.4 ^i^	
Total	43	19.8	15	6.9	159	73.3		45	20.8	28	13.0	143	66.2		81	37.5	16	7.4	119	55.1	

(**a**) NS = not significant. ^^^ Analysis excluded for Central and Eastern Europe (*n* = 9) region, and skill (*n* = 18) sport category due to low participant numbers. Chi-squared test statistics = ^a^ X^2^(2) = 8.30; ^b^ X^2^(6) = 14.20; ^c^ X^2^(6) = 24.29; ^d^ X^2^(6) = 34.41. Superscript letters indicate a significant difference between groups across columns after Bonferroni correction (*p* < 0.05). (**b**) NS = not significant. ^^^ Analysis excluded for Central and Eastern Europe (*n* = 9) region, and skill (*n* = 18) sport category due to low participant numbers. Chi-squared test statistics = ^e^ X^2^(6) = 17.91; ^f^ X^2^(6) = 30.80; ^g^ X^2^(2) = 8.83; ^h^ X^2^(2) = 23.24; ^i^ X^2^(2) = 9.41. Superscript letters indicate a significant difference between groups across columns after Bonferroni correction (*p* < 0.05).

**Table 6:** Characteristics of participants who completed the staff training survey (*n* = 310).

	Total		Male		Female		*p*
	*n*	%	*n*	%	*n*	%	
Sex			118	38.2	191	61.8	
Age							NS
29 years or younger	195	63.1	70	35.9	125	64.1	
30 years or older	114	36.9	48	42.1	66	57.9	
Region							NS
Africa	9	3.0	6	66.7	3	33.3	
Latin America and Caribbean	42	13.8	14	33.3	28	66.7	
Central and Eastern Europe	50	16.4	22	44.0	28	56.0	
Western ^^^	152	50.0	51	33.6	101	66.4	
Asia Pacific	51	16.8	22	43.1	29	56.9	
Role							0.003
Chef or cook	95	33.5	47	49.5 ^a^	48	50.5	
Food and beverage delivery	140	49.3	37	26.4	103	73.6 ^a^	
Manager/supervisor	19	6.7	6	31.6	13	68.4	
Other ^$^	30	10.6	13	43.3	17	56.7	
Level of education							NS
Certificate or less	141	48.1	56	39.7	85	60.3	
University undergraduate or higher	152	51.9	57	37.5	95	62.5	
Level of experience							NS
Up to 2 years	206	66.7	79	38.3	127	61.7	
More than 2 years	103	33.3	39	37.9	64	62.1	
Prior nutrition training							NS
Yes	74	23.9	27	36.5	47	63.5	
No	235	76.1	91	38.7	144	61.3	

Missing values for sex (*n* = 1), age (*n* = 1), region (*n* = 5), role (*n* = 26), level of education (*n* = 17), level of experience (*n* = 1), and prior nutrition training (*n* = 1). NS = not significant. ^^^ Western includes Australia, New Zealand, Canada, the United States, and the United Kingdom. ^$^ Role: “other” includes muster room attendants, event desk officers, and those unsure of their role. ^a^ Chi-squared test statistic (X^2^ = (3)13.86). Significant difference in the proportion of males/females between chefs/cooks and food and beverage delivery staff after Bonferroni correction (*p* < 0.05).

**Table 7:** Proportion of menu items that differed from the serving weight on the nutrition card.

Difference between Actual Weight and Serving Weight on Card	Number of Menu Items as Proportion of the Total
10–49% less on the card	10%
10–49% greater on the card	18%
50% less on the card	37%
50% greater on the card	18%

## Data Availability

Not applicable.

## Supplementary Materials

The following supporting information can be downloaded at: https://www.mdpi.com/article/10.3390/nu15214678/s1, Table S1: Quality assurance comments.
