# Supplementary material for: Evaluation of an Environmental Nutrition Intervention at the 2018 Commonwealth Games
Source: Nutrients. 2023 Nov 4;15(21):4678. doi: 10.3390/nu15214678 (PMC10647693; doi:10.3390/nu15214678)
Supplement: Supplementary file 1 [file nutrients-15-04678-s001.zip › nutrients-2630623-supplementary.pdf]

Table S1; Quality assurance comments

Comments on the quality of the food, proportions presented per theme.

Theme		Positive comments	n (%)	Neutral comments	n (%)	Negative comments	n (%)
Sensory properties n=560	Taste	"tastes nice" (kangaroo pie, orange almond cake) "very tasty" (jerk chicken, beef kebab)	130 (23.2)	"tasted fine" (buttermilk pancake) "tastes ok" (black pudding) "tastes sweet" (potato and pumpkin curry, ketchup chicken, waffles, Rajmah masala)	59 (10.5)	"Really disgusting after taste. Don't know if this is how it is meant to taste." (Plain congee) "Bland" (sauteed mushroom, stir fry veg, noodles, hollow rice, coleslaw, salad, chickpea burger, African buffalo meat stew halal)	47 (8.4)
	Appearance	"looks appealing" (waffles, angus rump steak, sauteed mushroom) "Looks nice and fresh" (assorted steam veg, steamed broc, green bean, fruit salad, Margaretta pizza, meat lovers pizza, salad, bone broth, okra with tomato gumbo)	90 (16.1)	"looks as expected" (porridge, pork schnitzel) "looked ok" (grilled chicken breast)	29 (5.1)	"looks a little shrivelled up" (Steamed greens) "Snow peas looked really cooked and just like the ones they had on for hot service last night" (rice noodle salad)	29 (5.1)
	Texture	"Not chewy, nice and crunchy batter" Chicken Schnitzel "Not too chewy" (Slow cooked beef ragout)	74 (13.2)	"Pastry texture, quite crunchy and then chewy" (Roti) "Sauce is thick" (house made bake beans)	27 (4.8)	"Very tough, dry and chewy" (Smoked sliced biltong)	43 (7.7)
	Smell	"Smells great" (jerk chicken) "Smells nice" (Lamb chickpea and spinach curry) "good smell" (Soy braised pork stew)	16 (2.8)	"strong Banana Smell" (banana smoothie) "No smell" (Nasi Goreng) "Smells like coconut" (Coconut vegetable curry)	10 (1.8)		0 (0)
	Temperature	"temperature good" (Spinach and ricotta cannelloni)	1 (0.1)	"temperatures ok" (chicken kebab)	1 (0.1)	"Dry but could be due to meat being cold before eating" (Turkey Steak) "(may be due to tasting when dish is cold)" (Honey roasted pumpkin)	4 (0.7)

Food preparation n=165	Cooking technique	"well cooked" (buttermilk pancake, jerk chicken, African smoked fish, bacon, grilled barramundi, chicken breast)	45 (27.2)	"Very tough, dry and chewy but nicely smoked" (Smoked sliced biltong)<br>"It looks and tastes fried, Likely wrong card or wrong item" (Grilled chicken breast)	2 (1.2)	"slightly undercooked" (buttermilk pancake)<br>"overcooked" (kangaroo, calamari)	26 (15.7)
	Oil content of food	"not too oily" (pesto pasta, bacon, baked salmon, roasted root vegetable)	8 (4.8)	"I think coconut oil or coconut cream is on it?" (Fried plantain)<br>"Oil added not on card" (sweet potato)	2 (1.2)	"very oily" (Chicken chipolata, grilled chicken, roasted eggplant, roasted root vegetables, Sauteed potato, chicken rendang, chicken tikka masala)<br>"looks oily" (kangaroo sausage)	24 (14.5)
	Ingredient ratio/amount	"plenty of herbs and capsicum" (Egg white omelette)<br>"plenty of ingredients" (BBQ chicken, grilled capsicum, red onion and coriander pizza)<br>"I like that I can see the different vegetables inside." (Beef bourguignon with shallot)	8 (4.8)	"Contains whole egg" (quiche Lorraine)	1 (0.6)	"Would suggesting using one other colours vegetable, green capsicum was only used and looks a bit dull" (seafood kebab)<br>"small amount of Tim Tam smash" (Chocolate fudge cake with smashed Tim Tams)<br>"not much mixed in such as prawn, chicken, bean shoots, capsicum, basically just noodles" "Minimal toppings"	49 (29.7)
Service n=14	Plating		0 (0)		0 (0)	"Large scoop but no mince was plated. Whether the utensil is incorrect or training needs to be done forge staff" (Shepards pie)<br>"staff didn't know how much was okay to give" (Roast pork)<br>"not sliced" (Smoked mackerel)<br>"only provided 1 wedge not 2" (Boiled coco yam)<br>"If serving size was correctly given it would be the right household size" (steamed green beans)	8 (57.1)

	Sitting time		0 (0)		0 (0)	“Sitting in Bain for a while” (Angus beef steak, grilled chick) “sitting for a little too long” (assorted veg) “Needed to be stirred as it was splitting.” (miso soup) “separated” (meen mouile, chicken rendang)	6 (42.9)
Total n=739			372 (50.3)		131 (17.7)		236 (32.3)

Counts and sample comments about aspects of the nutrition label, row proportions presented and column proportion for incorrect category only.

	Correct		Neutral		Incorrect		
**Component**	**Comment**	**Row n (%)**	**Comment**	**Row n (%)**	**Comment**	**Row n (%)**	**Column %**
Title n=29	"is true to nutrition card" (house made baked beans)	3 (10.3)	"close to card" (stir fry veg)	1 (3.4)	"Tastes like canned baked beans with added kidney beans. Probably should just be called baked beans" (house made baked beans)	25 (86.2)	6.6%
Size n=136	"For this serve size it looked to be about a cup" (roast root vegetables) "Yes, 3 kernels were provided" (Baby corn kernels)	13 (9.6)	"1 roll served which was approx. correct weight but card says 2 rolls" (spinach and ricotta cannelloni)	8 (5.9)	"Removed amount to be half a cup and still weighed 114g" (Gari)	115 (84.6)	30.5%
Ingredient n=130	"is true to nutrition card and ingredients" (house made baked beans)	4 (3.1)	"Chilli, tomato, plantain, oil" (Plantain)	2 (1.5)	"No green apple, no cinnamon" (bircher museli) "No pepper crust" (pepper crusted salami) "Also contains baby corn and green beans in addition to what nutrition card states" (assorted steam vegetables)	124 (95.4)	32.9%
Energy and macronutrients n=58	"Oily on the plate but fat content high on card so probably reflective" (African chicken kebabs)	4 (6.9)	"As corrected to serving size" (Gnocchi with sage and vegetable and coconut chicken curry)	3 (5.2)	"Mine probably lower in protein as very little bacon on it" (Egg bacon and cheese muffin) "More fat due to extra oil" (grilled chicken)	51 (87.9)	13.5%
Symbols n=36	"No gluten containing foods listed on card but has gluten free symbol." (Jerk chicken)	3 (8.3)	"Seeds?" (bubble and squeak, egg white omelette)	3 (8.3)	"Should say it is vegetarian but does not" (Mac and cheese pasta bake) "Added corn - more energy dense" (assorted steamed vegetables)	30 (83.3)	8.0%
Sodium n=35	"Correct for 1roll" (Spinach and ricotta cannelloni) "Looks as nutrition card indicates" (Okra with tomato gumbo)	2 (5.7)	"close to card" (stir fry veg)	1 (2.9)	"Salt added to dish, listed as low sodium" (sautéed Kale and green beans)	32 (91.4)	8.5%

Total<br>n=424		30<br>(7.0)		17<br>(4.0)		377<br>(88.9)	100%
